# Impact of Bacterial Vaginosis, as Assessed by Nugent Criteria and Hormonal Status on Glycosidases and Lectin Binding in Cervicovaginal Lavage Samples

**DOI:** 10.1371/journal.pone.0127091

**Published:** 2015-05-26

**Authors:** Bernard J. Moncla, Catherine A. Chappell, Lara K. Mahal, Brian M Debo, Leslie A Meyn, Sharon L. Hillier

**Affiliations:** 1 Department of Obstetrics, Gynecology and Reproductive Sciences, University of Pittsburgh, Pittsburgh, Pennsylvania, United States of America; 2 Magee-Womens Research Institute, Pittsburgh, Pennsylvania, United States of America; 3 Biomedical Chemistry Institute, Department of Chemistry, New York University, New York, New York, United States of America; 4 Department of Cell and Developmental Biology, University of Pennsylvania, Philadelphia, Pennsylvania, United States of America; Fred Hutchinson Cancer Center, UNITED STATES

## Abstract

The objective of this study was to evaluate the impact of hormonal status and bacterial vaginosis (BV) on the glycosidases present and glycosylation changes as assessed by lectin binding to cervicovaginal lavage constituents. Frozen cervicovaginal lavage samples from a completed study examining the impact of reproductive hormones on the physicochemical properties of vaginal fluid were utilized for the present study. In the parent study, 165 women were characterized as having BV, intermediate or normal microflora using the Nugent criteria. The presence of glycosidases in the samples was determined using quantitative 4-methyl-umbelliferone based assays, and glycosylation was assessed using enzyme linked lectin assays (ELLA). Women with BV had elevated sialidase, α-galactosidase, β-galactosidase and α-glucosidase activities compared to intermediate or normal women (*P*<0.001, 0.003, 0.006 and 0.042 respectively). The amount of sialic acid (*Sambucus nigra*, *P* = 0.003) and high mannose (griffithsin, *P*<0.001) were reduced, as evaluated by lectin binding, in women with BV. When the data were stratified according to hormonal status, α-glucosidase and griffithsin binding were decreased among postmenopausal women (*P*<0.02) when compared to premenopausal groups. These data suggest that both hormonal status and BV impact the glycosidases and lectin binding sites present in vaginal fluid. The sialidases present at increased levels in women with BV likely reduce the number of sialic acid binding sites. Other enzymes likely reduce griffithsin binding. The alterations in the glycosidase content, high mannose and sialic acid binding sites in the cervicovaginal fluid associated with bacterial vaginosis may impact susceptibility to viruses, such as HIV, that utilize glycans as a portal of entry.

## Introduction

Secreted cervical mucus coats the vaginal and cervical epithelium forming a protective biochemical and physical barrier to infection. The mucus is composed of mucins, glycoproteins and other secreted proteins, including antibodies, antibacterial proteins and peptides. Secreted mucins, the gel-forming component of mucus, are large, highly glycosylated proteins (10–40 MDa) that form a viscoelastic gel [1–5]. Branched carbohydrate chains (3–10 sugars in length) consisting of N-acetyl-glucosamine, N-acetyl-galactosamine, galactose, fucose and sialic acid account for up to 80% of the weight of mucins [6, 7].

Bacterial vaginosis (BV) is a common vaginal syndrome in which lactobacilli are replaced by a diverse community of anaerobic and facultative microorganisms [8–10]. Microorganisms recovered from the vagina of women having BV produce a range of glycosidases, including sialidase, and the presence of these microorganisms is associated with decreased vaginal fluid viscosity and increased symptoms of vaginal discharge [11, 12]; however, to date, no one has quantified the glycosidases in cervicovaginal lavage (CVL) components and concurrently evaluated changes in lectin binding.

Sialidases (neuraminidases), which cleave α-ketosidic linkages between the glycosyl- residues of glycoproteins, glycolipids, or colominic acids and sialic acid, are found in both mammalian and bacterial systems. Sialidase is considered to be a virulence factor in many pathogenic bacteria which infect mucosal surfaces, including: *Prevotella*, *Bacteroides*, *Porphyromonas*, *Corynebacterium diphtheriae*, *Vibrio cholerae*, *Streptococcus pneumoniae*, *Gardnerella vaginalis and* group B streptococci [11, 13–17].

HIV glycoprotein 120 is highly glycosylated with terminal sialic acid linked α-2-6-galactose [18]. The HIV receptor on CD4 cells, the primary T lymphocyte population in the genital tract associated with transmission of HIV is also glycosylated with terminal sialic acid residues in α-2-3 linkages. The treatment of cells or HIV with sialidase (neuraminidases) alters the dynamic of infection to the advantage of the virus, but the mechanism is unknown [19–21].

There is a limited understanding of the human vs microbial glycosidases and mucinases present on the surface of the vagina and cervix [21], and the impact of reproductive hormones on these is unexplored. Cauci et al. presented evidence that endogenous proteases cleave antibodies in the genital tract of pregnant women, and Lewis et al. have demonstrated that the addition of exogenous glycosidases and protease alters sIgA [22–24] in the vaginal fluid of reproductive age women. It is largely assumed that the glycosidases measured in the cervicovaginal fluid of women with BV are of bacterial origin, but there is no direct demonstration of the effects of the glycosidases on the glycoproteins of the female reproductive tract. We now demonstrate that in women with BV, there is an increase in four glycosidases, suggesting that these are correlated with a change in the bacterial flora. The increase in glycosidases in vaginal fluid of women with BV is associated with a concomitant decrease in lectins binding to both high mannose (griffithsin) and α-2, 6 sialic acid (SNA). Our data suggests that changes in glycosidases are accompanied by changes in glycosylation patterns in the vaginal fluid. Post-menopausal women also had decreased high mannose binding, suggesting that reproductive hormones may also impact glycosylation patterns.

## Materials and Methods

### Study Population

This was a secondary analysis of samples collected as part of a study of the physical properties of vaginal fluid, and the complete methods describing the study populations are described elsewhere [25]. Written informed consent was obtained following a protocol approved by the University of Pittsburgh IRB. Women were excluded if they were: breastfeeding or pregnant; presented vaginal symptoms including vaginal discharge, pruritus, malodor, or vulvar/vaginal burning; with any cervical or vaginal infections or had used any antimicrobials in the past 14 days; had used any vaginal devices or vaginally-applied products (excluding tampons) in the past week. Upon enrollment the women had: an OraQuick advance rapid HIV test; a pregnancy test; their demographic information recorded; height and weight taken and medical, gynecologic and sexual histories taken. Cervicovaginal lavage (CVL) was collected from 165 women characterized as: post-menopausal; first 14 days of cycle, (1–14 days of menstrual cycle); second 14 days of cycle, (15–30 days of menstrual cycle); oral contraceptives; depo- medroxyprogesterone acetate (DMPA); or women using the Mirena intrauterine device (IUD). Vaginal smears were Gram stained and evaluated using the Nugent criteria [26].

### Sample Collection

CVLs were collected in 10 mL of sterile normal saline (Hospira, Inc. Lake Forest, IL 60045). The saline and a syringe were used to gently wash the ectocervix and vaginal vault for 1 minute and stored on ice until the fluid was transported to the laboratory within 60 minutes.

### CVL processing

Upon receipt in the laboratory, CVLs were dispensed into 2 mL cryovials. Samples received 10μL/mL of protease inhibitor (Sigma-Aldrich) and the samples were stored at -80°C.

### Enzyme-Linked Lectin Assays (ELLA)

CVLs were diluted to give a final SDS (Sigma-Aldrich, St. Louis MO) concentration of 1% in 50mM sodium carbonate buffer. The samples were heated in a boiling water bath for 5 min added to flat bottom 96 well clear microtiter plates (Nunc, Thermo Fisher, 75, Panorama Creek Dr., Rochester, NY 14625), 300–500 ng protein /100μL and 50μL added per well and allowed to air dry in an oven set to 40°C overnight. Plates were washed 4 times in phosphate buffered saline (PBS), blocking buffer was added (PBS plus 0.5% polyvinyl alcohol 30–50 KDa (Sigma-Aldrich, St. Louis, MO) and incubated at room temperature for 1 hr. Plates were washed 4 x with PBS. Horseradish peroxidase labeled lectins (Vector Laboratories, Burlingame, CA) were diluted to the optimum concentration in PBS containing 0.5% PVA and 0.05% Tween 20 and 50 μL added to each well. Plates were covered with aluminum foil and incubated on orbital shaker at setting (medium speed), and incubated for 1 hour. Plates were washed 4 X with 0.05% Tween 20 in PBS. The plates were developed using 3,3′,5,5′-Tetramethylbenzidine (TMB) Liquid Substrate System for ELISA (Sigma-Aldrich, St. Louis, MO,) and incubated for an appropriate time. The reactions were stopped by the addition of 50 μL 1N H_2_SO_4_ and the optical densities read with a DTX880 Multimode Detector (Beckman-Colter, Fullerton, CA). Each plate included dilutions of MCF7 cell lysate Santa Cruz Inc. Santa Cruz, CA.) as mucin standards and an appropriate dilution of cervical mucus that was a pool of four different women (Lee Bio-Solutions, St. Louis, MO). ELISA data are presented as ng/ng protein.

Protein concentrations of the CVL were determined using a modification of Lowry [27, 28] since other methods are known to give unreliable values for glycoproteins. Previous studies have reported their results in units measured per volume using extrapolation to calculate the concentration in the CVL [29–31]. This method assumes that the samples are equivalent with respect to dilution. The range of protein concentrations in our samples was: 0.23 mg/mL to 6.11 mg/mL, a 26 fold range. This range may reflect variable dilution of the vaginal fluid and mucus during sample collection and expected variability in protein content due to the many changes that occur in the female cycle in response to hormones as well as differences in the water and ion content [29, 32–38]. Therefore, protein concentration was used to standardize the samples.

### Glycosidase assays

Glycosidase assays were performed by fluorescence, in microtiter plates using a modification of the methods in Moncla et al. [15, 39]. Briefly, 4-methyllumbelliferyl- sugar derivatives (Sigma-Aldrich, St. Louis, MO) (-α-D-glucoside, β-D-glucoside, α-L-fucoside, β-D-fucoside, α-D-galactoside, β-D-galactoside, and α-D-N-acetyl neuraminic acid) (Sigma-Aldrich, St. Louis, MO) were suspended in ultra-pure water Milli-Q (Millipore, Billerica, MA) to a final concentration of 0.2 mM. Samples were incubated for 20 min at room temperature and stopped by the addition of 0.1M borate buffer pH 9.2 and read in a DTX880 Multimode Detector (Beckman-Coulter, Fullerton, CA), excitation light 365 emission 450.

## Results and Discussion

### Impact of vaginal flora and BV on glycosidases and glycosylation patterns

In women, mucus is secreted at the cervix and exposed to sialidases and other glycosidases of host or bacterial origin. In order to assess the possible role of bacterial glycosidases, in modulating the glycome in the vaginal vault, the Nugent score was used to characterize the vaginal microflora as *Lactobacillus*-predominant, intermediate or consistent with BV. Post-menopausal women are excluded from these analyses because the Nugent score has only been validated for use in women of reproductive age. When we stratified the data by glycosidases and lectin binding in CVL across the three Nugent categories, we observed differences in both glycosidases activities and glycan pattern. As shown in Table 1, several significant associations were observed. Increased levels of sialidase, β-galactosidase and α-glucosidase activity were detected in the CVLs of women with BV (*P* = <0.001, 0.006, 0.042, respectively). Conversely, α-galactosidase activity was significantly decreased in women with BV compared with normal women (*P* = 0.04). There was no association between Nugent score and α-fucosidase or β-glucosidase activity.

**Table 1:** Enzymatic activities and lectin binding in the cervicovaginal fluid of premenopausal women, stratified by Nugent scores.

	Nugent Score			* *
	Normal (n = 89)	Intermediate (n = 23)	Bacterial Vaginosis (n = 23)	*P-value* ^*2*^
	Nugent score 0–3	Nugent score 4–6	Nugent score 7–10	
**Glycosidases**				
** Sialidase**	0 (0–6.90)	0 (0–6.21)	3.88 (0–9.85)	<0.001
** α-fucosidase**	0.33 (0–2.64)	0.33 (0–0.97)	0.29 (0–1.31)	0.83
** α-galactosidase**	0.31 (0–4.79)^4^	0 (0–2.50)	0.19 (0–3.47)^4^	0.003
** β-galactosidase**	0 (0–23.84)	0 (0–2.12)	0.17 (0–1.73)	0.006
** α-glucosidase**	5.91 (0–67.02)	8.59 (0–41.21)	14.31 (0–61.27)	0.042
** β-glucosidase**	0 (0–3.96)	0 (0–0.91)	0 (0–0.45)	0.23
**Lectin binding**				
** Maakia amurensis**	0.15 (0.07–0.56)	0.14 (0.11–0.28)	0.13 (0.08–0.31)	0.28
** Sambucus nigra**	0.16 (0.05–0.81)	0.19 (0.08–0.33)	0.11 (0.06–0.20)	0.003
** Griffithsin** ^**1**^	10.36 (0–35.39)	8.48 (0.89–33.38)	3.14 (0.97–11.92)	<0.001

^1^Data are presented as medians (range) in μMoles substrate hydrolyzed per min per mg protein. Griffithsin is measured as pg/ng protein.

^2^ *P*-value from Kruskal-Wallis test.

^4^ *P*-value = 0.04 from Mann-Whitney U test comparing normal women to women with bacterial vaginosis for alpha galactosidase.

Numerous studies have noted the association between BV and increased sialidase activity [11, 21, 22, 40–42]. An increase in β-galactosidase activity associated with BV has been reported by Howe et al. However, we cannot directly compare our result to their report since they did not adjust their results by protein content of the lavage fluid [41]. The higher levels of α-glucosidase observed in the present study have not been reported previously, but could be due to the presence of organisms that produce this enzyme, for example *Gardnerella vaginalis* or *Prevotella* species [17, 43].

With the increased levels of sialidase activity in women with BV, we hypothesized that we would observe a reduction in sialic acid ligands in the CVL. Consistent with this hypothesis, we observed a significantly lower quantity of α-2, 6 sialic acids in the women with BV (*Sambucus nigra* SNA, Table 1). However, there was no association between BV and *Maakia amurensis* lectin (MAL) binding (*P* = 0.28, Table 1). The binding of MAL is specific for α-2, 3 linked terminal sialic acids while the (SNA) recognizes α-2, 6 bound sialic acids. However, MAL type II can bind both α-2, 3 sialylated and 3-O-sulfated glycans [44] which could explain why MAL type II observed binding did not differ in women with BV.

The binding of Griffithsin was greatly decreased in samples from women with abnormal flora (*P* <0.001) consistent with observations in our companion paper [45]. Because high-mannose is relevant to HIV binding to receptor cells, we also evaluated high-mannose binding sites in CVL with the algal lectin Griffithsin (GRFT). GRFT binding was also reduced in women with abnormal flora. Wang et al., using the same CVL samples, also observed a significantly reduced GRFT binding using a lectin array [45]. Further, in their assay, other lectins that recognize high mannose were also lower in women with BV. This reduction in GRFT binding sites could be the result of microbial or host enzymes [46] or could reflect changes in expression of the epitope.

The increase in sialidase levels in BV is well established. However, the present study is the first to associate higher sialidase levels in CVL samples directly with lower levels of sialylation of the proteins in that same sample. Since so many of the organisms associated with BV have been demonstrated to have sialidase, and other enzymes [11, 16, 39, 47] we assume the enzymes detected are of bacterial origin. The lower level of protein sialylation among with with BV is likely the result of bacterial sialidase activity. When proteins in CVL are measured by techniques such as ELISA which use antibodies to the peptides, alterations in sialylation will not be detected.

The changes in protein sialylation associated with BV are biologically relevant because the carbohydrate components of glycoproteins help to protect those proteins from degradation. When the negatively charged sialic acid is cleaved from the carbohydrate chain, the remaining sugars are susceptible to both endo- and exoglycosidases [48–50]. After the remaining carbohydrates are removed, the protein becomes susceptible to proteolysis. Recently, Lewis et al presented evidence that increased proteolysis occurs in vaginal fluid of women with BV. They evaluated the capacity of vaginal fluid to affect changes in immunoglobulins in an *in vitro* assay. When vaginal fluid was incubated with sIgA and sialidase, there was degradation of the sIgA [24]. Cauci et al. also noted the correlation of sialidase activity in CVL with immunoglobulin degradation [51].

The results of our study used CVL samples from women with or without BV (stratified by Nugent score or hormonal status respectively) and the levels of sialidase were measured. The reduced sialylation of proteins in the CVL as detected by the reduction in SNA binding in women with BV extend the findings of Lewis and Cauci by demonstrating that there is a good correlation between the increased level of sialidase in vaginal fluid and reduced sialylation of the proteins in that same sample.

The lectin binding data indicates the quantities of α-2,3 and α-2,6-linked sialic acids are comparable among the samples of women having a *Lactobacillus*-predominant vaginal microflora. *Prevotella bivia*, an obligately anaerobic microorganism associated with BV has a sialidase which is about 50% more active in cleaving α-2,6-linked sialic acid than α-2,3-linked sialic acid, which is consistent with the SNA signature of women with BV. The sialidase of *Prevotella bivia* is cell associated, whereas the sialidase of *Gardnerella vaginali*s is only produced by 25% of isolates and is secreted into the medium (B.J. Moncla unpublished observations). Given the many different organisms associated with BV, the sialidase and exoglycosidase activities in the vaginal milieu may play a role in the development of BV. Longitudinal studies of women before and after development of BV will be necessary to shed light on the role of these glycosidases on the pathogenesis of this syndrome.

### Impact of hormonal status and contraceptives on glycosidases and glycosylation patterns

Because of the changes in glycosidases and lectin binding observed in women with BV, we restricted our analysis of differences in these parameters among women having different hormonal status to those women who did not have BV. Recently we reported that CVLs from post-menopausal women have statistically significantly lower levels of protein compared to women of reproductive age [25]. Even when normalized by protein levels, post-menopausal women displayed lower levels of α-glucosidase (*P* = <0.02), griffithsin binding and higher SNA (α-2,6 linked sialic acids) binding than women of reproductive age. The lectin studies demonstrated higher binding of SNA in the premenopausal days 1–14 days of cycle group and among IUD users (Table 2). Comparing women in days 1–14 days of cycle with women in days 15–28 days we found the women in days 1–14 of cycle had higher α-glucosidase activity (*P* = 0.047) and greater binding of GRFT (*P* = 0.02) than women in days 15–28 of cycle. Thus, among the pre-menopausal women we observed very subtle changes in the level of the different carbohydrate display that appears to be controlled by the levels and types of hormones. Similar results were obtained, using a lectin microarray [45]. Most of the other enzymes measured in the CVL were not significantly different when stratified by hormonal group, with the exception of α-glucosidase. This enzyme was lowest in the post-menopausal group while the DMPA group had 8 fold higher levels than the post-menopausal group (Table 2). The elevated levels of α-glucosidase could reflect the presence of bacteria such as *G vaginalis* or *Lactobacillu*s spp. which can produce α-glucosidase, The decrease in α-glucosidase in post-menopausal women is consistent with the decrease in *Lactobacillus* among these women. The decreases in sialic acid specific lectin binding suggest that the sialidases are active against the glycoproteins in the female reproductive tract. By extension the other enzymes detected and associated with BV are most likely as active against the glycoproteins as the sialidase. Our observations of increased sialidase activity are consistent with the work of Wang et al. who reported an increase in signals for terminal β- galactosides and β- N-acetylgalactosides [45]. Since cleavage of sialic acid would reveal these epitopes. Thus, there are several factors controlling the lectin-glycan interactions. Work with *Streptococcus* spp. and other organisms has demonstrated that exoglycosidases have substrate specificities similar to those we described for sialidase in the present study; further these enzymes are important in both promoting colonization and infection [21, 52, 53]. King et al. elegantly demonstrated the sequential hydrolytic attack of human glycoproteins by exoglycosidases produced by streptococci [53].

**Table 2:** Median levels of glycosidases and lectin binding stratified by hormonal group, excluding women with bacterial vaginosis.

	Postmenopausal (n = 29)	Premenopausal, Women in days 1–14 of menstrual cycle (n = 19)	Premenopausal, Women in days 15–28 of menstrual cycle (n = 23)	Oral Contraceptives (n = 24)	Depot medroxyprogesterone acetate (n = 19)	Mirena Intrauterine Device (n = 25)	*P*-value^2^
**Glycosidases**							
** Sialidase**	0 (0–4.89)	0 (0–6.21)	0 (0–6.90)	0 (0–0.07)	0 (0–0.22)	0 (0–1.16)	0.02
** α-fucosidase**	0.36 (0–1.15)	0.41 (0.02–0.94)	0.29 (0–1.14)	0.29 (0–0.84)	0.37 (0–0.93)	0.37 (0–2.64)	0.74
** α-galactosidase**	0 (0–1.25)	0.01 (0–2.61)	0 (0–4.34)	0 (0–4.79)	0 (0–3.11)	0 (0–2.76)	0.88
** β-galactosidase**	0 (0–7.34)	0.01 (0–2.03)	0 (0–23.84)	0 (0–3.84)	0 (0–2.72)	0 (0–2.46)	0.86
** α-glucosidase**	1.23 (0–59.37) ^3^	7.90 (0.03–67.02)	4.47 (0–25.80)^4^	7.07 (0–48.04)	8.16 (0–28.23)	5.98 (0–41.21)	0.01
** β-glucosidase**	0 (0–5.00)	0 (0–0.18)	0 (0–3.96)	0 (0–0.14)	0 (0–0.23)	0 (0–0.91)	0.28
**Lectin binding**							
** *Maakia amurensis***	0.16 (0.07–0.28)	0.18 (0.09–0.35)	0.14 (0.09–0.56)	0.16 (0.07–0.38)	0.19 (0.10–0.36)	0.14 (0.09–0.25)	0.04
** *Sambucus nigra***	0.23 (0.08–1.18) ^3^	0.18 (0.09–0.28)	0.14 (0.05–0.81)	0.15 (0.07–0.34)	0.14 (0.06–0.62)	0.17 (0.08–0.33)	0.24
** Griffithsin** ^**1**^	4.75 (0.77–20.20)	10.80 (1.32–20.35)	6.89 (2.22–18.06) ^4^	11.57 (0–35.39)	10.97 (0.89–33.38)	10.30 (1.28–23.29)	0.001

^1^Data are presented as medians (range) in μMoles substrate hydrolyzed per min per mg protein. Griffithsin is measured as pg/ng protein.

^2^ *P*-value from Kruskal-Wallis test across all groups.

^3^ *P*-value <0.02 from Mann-Whitney U test comparing post-menopausal women to women of reproductive age.

^4^ *P*-value = 0.02 and 0.047 from Mann-Whitney U test comparing women in days 1–14 of cycle to women in days 15–28 of the cycle for, respectively Griffithsin and α-glucosidase.

## Conclusions

In conclusion we have demonstrated that in women with BV, there is an increased levels of in four of 6 glycosidases studied, suggesting that these are of bacterial origin. We also made the novel observation of a correlation of BV with α-glucosidase. The increase in glycosidases in vaginal fluid of women with BV is associated with a concomitant decrease in lectins binding, both high mannose (griffithsin) and α-2, 6- sialic acid (SNA). These data suggest that changes in glycosidases are accompanied by changes in glycosylation patterns in the vaginal fluid. These data suggest that the vaginal microflora can have a much greater impact on the overall vaginal environment than do hormones. Women not using exogenous contraceptive hormones demonstrated changes in α-glucosidase activity and GRFT binding in the second 14 days of the cycle compared to the first 14 days of the cycle. Post-menopausal women also had decreased high mannose binding, illustrating how reproductive hormones impact glycosylation patterns. These changes may result in the increase risk of HIV infection among women with BV.

## Supporting Information

S1 Table Reagents used for the work presented. (DOCX)

S2 Table Primary data used to derive the data presented. (XLSX)
