# Supplementary material for: Impact of Bacterial Vaginosis, as Assessed by Nugent Criteria and Hormonal Status on Glycosidases and Lectin Binding in Cervicovaginal Lavage Samples
Source: PLoS One. 2015 May 26;10(5):e0127091. doi: 10.1371/journal.pone.0127091 (PMC4444347; doi:10.1371/journal.pone.0127091)
Supplement: S1 Table — (DOCX) [file pone.0127091.s001.docx]

**S1 Table. Reagents used for the work presented.**

|  |  |  | **Conjugation** | **primary/secondary** |
| --- | --- | --- | --- | --- |
|  | **Lot Number** | **Ab description / catalogue number ^1^** |  |  |
|  |  |  |  |  |
|  | **GR156588-3** | **Anti-MUC1 antibody [HMFG1 (aka 1.10.F3)] (ab70475)** | **none** | **primary** |
|  |  |  |  |  |
|  | **GR84514** | **Anti-MUC4 antibody (ab60720)** | **none** | **primary** |
|  |  |  |  |  |
|  | **GR176272-1** | **Anti-MUC7 antibody (ab55542)** | **none** | **primary** |
|  | **GR77955** | **Anti-Mucin 5AC antibody [1-13M1] (ab24070)** | **none** | **primary** |
|  |  |  |  |  |
|  | **07065-8C11** | **MUC7 Antibody 0.05 ml (H00004589-A01)** | **GST tag** | **primary** |
|  | **GR14922-6** | **Donkey Anti-Goat IgG H&L (HRP) preadsorbed (ab97120)** | **HRP** | **secondary** |
|  | **GR6560-7** | **Donkey Anti-Rabbit IgG H&L (HRP) (ab97064)** | **HRP** | **secondary** |
|  | **GR68321-4** | **Goat Anti-Human IgG Fc (HRP) (ab97225)** | **HRP** | **secondary** |
|  | **GR129315-6** | **Goat Anti-Mouse IgG H&L (HRP) preadsorbed (ab97040)** | **HRP** | **secondary** |
|  | **GR33627-1** | **Rabbit Anti-Goat IgG H&L (HRP) preadsorbed (ab97105)** | **HRP** | **secondary** |
|  | **GR8317** | **Rabbit Anti-Mouse IgG H&L (HRP) (ab6728)** | **HRP** | **secondary** |
|  | **GR87741** | **Rabbit Anti-Mouse IgG H&L (HRP) (ab97046)** | **HRP** | **secondary** |

**B-1265 *Maakia amurensis* lectin type II Biotinylated**

**B-1305 *Sambucus nigra* lectin Biotinylated**

**Griffithsin and anti-griffithsin antibody were a generous gift of Dr. Alex Cole, University of Central Florida.**

**^1^Antibodies with lot numbers beginning with G were obtained from abcam®, beginning with zero were obtained from Novis Biologicals. All lectins were obtained from Vector Laboratories.**
